# Six-year changes in the prevalence of obesity and obesity-related diseases in Northeastern China from 2007 to 2013

**DOI:** 10.1038/srep41518

**Published:** 2017-01-27

**Authors:** Jing Wu, Hongqin Xu, Xiuting He, Yi Yuan, Chunyan Wang, Jie Sun, Shumei He, Junqi Niu

**Affiliations:** 1 Department of Gerontology, the First Hospital attached to Jilin University, Xinmin Street, Changchun 130021, China; 2 Department of Hepatology, the First Hospital attached to Jilin University, Xinmin Street, Changchun 130021, China; 3 Department of Rheumatology and Immunology, the First Hospital attached to Jilin University, Xinmin Street, Changchun 130021, China

## Abstract

Obesity and obesity-related diseases are important public health challenges. In this study, we aimed to provide updated trends in the prevalence of these conditions. We conducted two independent cross-sectional surveys of the general population aged 20–75 years in 2007 and 2013 in Jilin, China. A total of 3636 (1719 males) and 1359 (602 males) participants were enrolled in the 2007 and 2013 surveys, respectively. Obesity-related diseases were defined as type 2 diabetes, hypertension, dyslipidemia and non-alcoholic fatty liver disease (NAFLD). The age-standardized prevalence of obesity, overweight, diabetes, pre-diabetes, dyslipidemia and NAFLD increased from 2007 to 2013 from 15.82% to 19.41%, 35.85% to 41.80%, 6.37% to 9.23%, 16.77% to 23.49%., 53.46% to 65.50%, and 23.48% to 44.31% in males, respectively, and from 13.18% to 18.77%, 31.11% to 37.54%, 4.41% to 8.48%, 8.10% to 16.49%, 41.96% to 54.70%, and 17.56% to 43.06% in females, respectively. However, the prevalence of hypertension remained stable (males: 38.10% vs. 38.63% and females: 33.04% vs. 33.01% in 2007 and 2013, respectively). The prevalence of obesity and obesity-related diseases, except for hypertension, increased significantly in the general population in Northeastern China. More targeted measures should be implemented to address the serious challenges presented by these diseases.

Obesity is a significant global health challenge, and its increasing prevalence has been considered a global pandemic, affecting countries worldwide including China^1^,^2^. The global prevalence of obesity in 2030 has been projected to be 1.12 billion^3^. Furthermore, obesity and overweight were estimated to have caused 3.4 million deaths in 2010^4^. Obesity is associated with a number of health issues, ranging from specific diseases such as type 2 diabetes, dyslipidemia, hypertension and non-alcoholic fatty liver disease (NAFLD) to reduced quality of life, psychosocial disturbances, decreased life expectancy, and increased economic burden^5^. The morbidity and mortality of obesity-related diseases can be reduced by maintaining strict control of obesity, and this approach should therefore be emphasized.

China is a large developing country, and the rapid economy development has led to changes in lifestyle, such as in dietary habits and physical activity; for example, meat consumption has increased drastically, vegetable and fruit intake has decreased slightly, and levels of physical activity have also been reduced^6^. Many studies have evaluated the prevalence of obesity and obesity-related diseases in China^2^,^7^,^8^,^9^,^10^,^11^,^12^, but data on the recent prevalence trends of these diseases in China are rare^13^,^14^,^15^,^16^,^17^,^18^,^19^. Jilin Province which located in northeast China has a population of approximately 27 million^20^. In 2007, we conducted a study to screen for the prevalence of obesity and obesity-related diseases (diabetes, hypertension, dyslipidemia and NAFLD) in Dehui City, which is representative of Jilin Province. Six years later, the prevalence of these diseases was reassessed. Our study aimed to assess the changes in the prevalence of obesity, diabetes, hypertension, dyslipidemia and NAFLD in China from 2007 to 2013 and provides evidence for health care providers to effectively address the challenges presented by obesity and obesity-related diseases.

## Results

### Characteristics of Chinese adults in Dehui aged 20–75 years in the 2007 and 2013 surveys

As shown in Table 1, a total of 3636 (1719 male) and 1359 subjects (602 male) completed the surveys in 2007 and 2013, respectively. All participants were of Han origin. The mean age of the participants was 45 years (36, 55) in 2007 and significantly increased to 52 years (44, 60) in 2013, whereas the sex composition did not differ. The proportion of residents engaged in agricultural work markedly decreased in 2013 compared with 2007. BMI, WC, SBP, DBP, and TC, TG, fasting blood glucose (FBG), alanine aminotransferase (ALT) and aspartate aminotransferase (AST) levels were much higher in 2013 survey than in the 2007 survey (P < 0.05). However, LDL-C and HDL-C were significantly lower in 2013.

In view of different prevalence between female and male, we divided subjects by gender in the following analyses. Age, BMI, SBP, TC, LDL-C, TG, HDL-C, FBG and AST in 2007 and 2013 maintained significant differences in both genders. Women had significantly higher WC and ALT levels in 2013 (P < 0.01); however, the difference in men was not significant. DBP in both genders did not significantly change between the two surveys (Table 2).

### Prevalence of obesity and obesity-related diseases

Table 3 shows the crude and age-standardized prevalence rates of obesity and obesity-related diseases among males and females in the two surveys. Compared with 2007, the age-standardized prevalences of obesity, overweight, diabetes, pre-diabetes, dyslipidemia and NAFLD increased by 3.59%, 5.95%, 2.86%, 6.72%, 12.04% and 20.83% in males respectively and 5.59%, 6.43%, 4.07%, 8.39%, 12.74% and 25.50% in females respectively in 2013. On the other hand, the prevalence of hypertension did not change significantly for either gender. Males had a higher prevalence of obesity and obesity-related diseases (hypertension, diabetes, pre-diabetes, obesity, overweight, dyslipidemia and NAFLD) than females in both 2007 and 2013.

**Figure 1:**
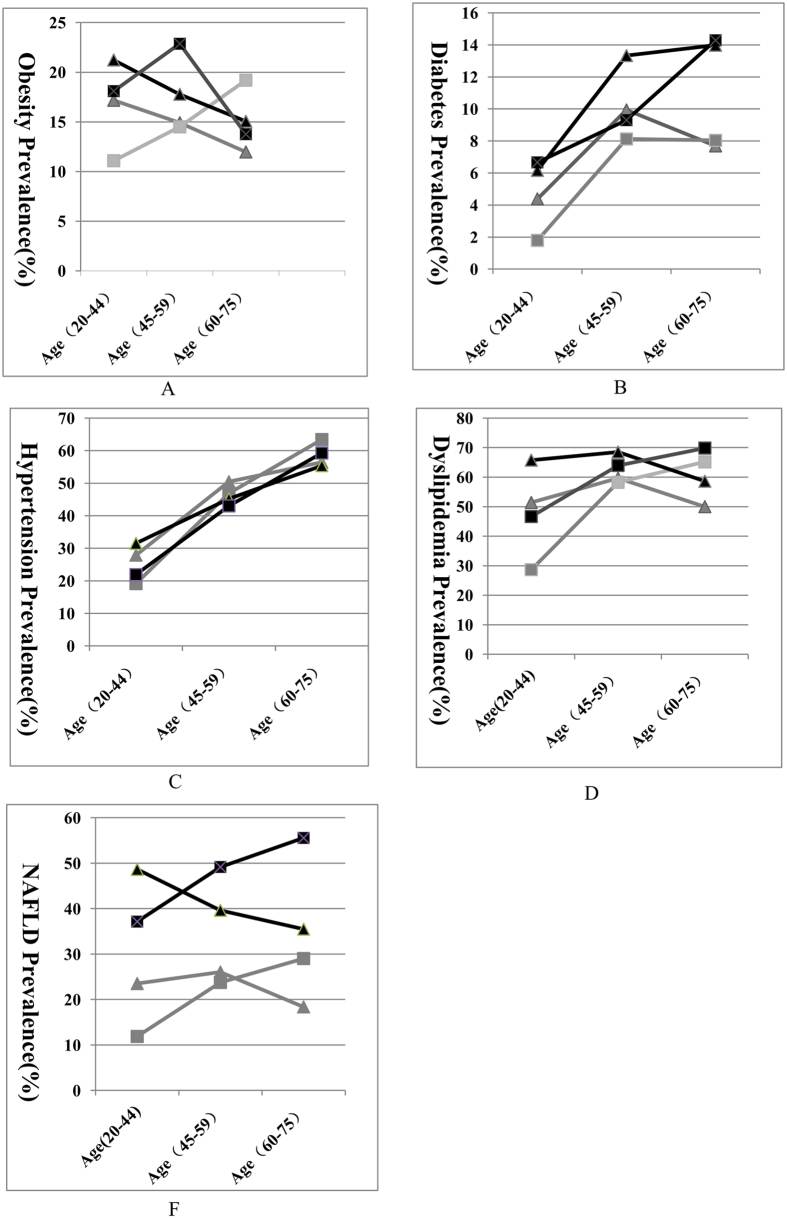
The prevalence of obesity and obesity-related diseases in different age groups. NAFLD, non-alcoholic fatty liver diseases; (**A**) Obesity prevalence; (**B**) Diabetes prevalence; (**C**) Hypertension prevalence; (**D**) Dyslipidemia prevalence; (**E**) NAFLD prevalence. The black line means 2013 years; the grey line means 2007 years; The triangle blot means males while the square blot means females.

The prevalences of obesity and obesity-related diseases stratified by the three age groups according to the above criteria for age-standardization^21^ are shown in Fig. 1. Obesity (Fig. 1A): In males, the prevalence of obesity decreased with age in the two surveys. In females, the prevalence of obesity increased with age in 2007, but in 2013, the highest prevalence was in the age range of 45–59 years, not 60–75 years. Diabetes (Fig. 1B): In 2007, the highest prevalence of diabetes occurred in the 45- to 59-year-old age group in both genders. However, in 2013, the prevalence showed increasing trends with age in both males and females. Hypertension (Fig. 1C): The overall prevalence increased with age in males and females in the two surveys. Dyslipidemia (Fig. 1D): In the two surveys, the highest prevalence among males was in those aged 45–59 years, and the lowest was in males 60–75 years; however, the prevalence increased with age in females. NAFLD (Fig. 1E): In females, the prevalence of NAFLD increased with age in both surveys, whereas middle-aged males tended to have more NAFLD.

The prevalence of dyslipidemia components is shown in Table 4. The age-standardized prevalence of high TG and low HDL-C levels increased significantly, but the prevalence of high LDL-C decreased significantly in both genders in 2013 compared with 2007. The prevalence of high TC decreased by 1.14% in men but increased by 4.07% in women from the 2007 to the 2013 survey.

## Discussion

Two independent cross-sectional surveys were conducted with participants aged 20–75 years in the same district (Dehui, Jilin, China) and with the same methods in both 2007 and 2013. In this study, the changes in obesity and obesity-related diseases prevalence in Dehui were explored. The age-standardized prevalence of obesity, overweight, diabetes, pre-diabetes, dyslipidemia and NAFLD increased significantly in both males and females from 2007 to 2013, whereas the age-standardized prevalence of hypertension did not significantly change.

Globally, the prevalence of obesity increased 1.9-fold (6.4% in 1980 vs. 12% in 2008)^22^ from 1980 to 2013, and the prevalence of overweight and obesity combined rose 47.1% among adults during the same period^1^. The trends in the prevalence of obesity and overweight in our study were consistent with those identified in previous studies. Obesity has a complex etiology, resulting from the combined effects of genetic, environmental, and lifestyle factors and the interactions between them^23^. Although genetic background is crucial to explaining individuals’ susceptibility to most chronic diseases, changes in lifestyle, including rapid urbanization, increased consumption of high energy density foods, and parallel decreases in physical activity, are considered the most likely factors contributing to this increase^23^,^24^,^25^. Under these circumstances, individuals develop high rates of obesity and obesity-related diseases such as type 2 diabetes, dyslipidemia and NAFLD^1^,^23^,^26^,^27^,^28^.

The prevalence of type 2 diabetes mellitus was estimated to be 21.5% in the 2002 World Health Organization (WHO) STEPwise approach to Surveillance (STEPS) survey; however, the corresponding prevalence in the 2013 STEPS survey was reported to be 45.8%^29^. The prevalence of diabetes in the US population increased from 5.5% to 9.3% from 1988–1994 to 1999–2010^30^. In Shanghai, China, the overall prevalence of diabetes increased from 27.93% to 34.78% between 2002 and 2012 in subjects with known risk factors for diabetes, such as a family history of diabetes, overweight or obesity, previously identified impaired fasting glucose or impaired glucose tolerance, history of gestational diabetes, polycystic ovary syndrome, hypertension, and dyslipidemia^31^. The global age-standardized diabetes prevalence increased from 4.3% in 1980 to 9.0% in 2014 in men and from 5.0% to 7.9% in women^32^. Although the contribution of each factor to the increased diabetes incidence cannot be discerned, the increase in diabetes overlaps with the increase in obesity in other studies^29^,^33^,^34^. Therefore, it is not surprising that the prevalence of diabetes and pre-diabetes increased over the 6-year period in our study.

In real-life settings, the management of dyslipidemia remains far from optimal, both in primary and secondary prevention^35^. Data from a Beijing adult population showed that the prevalence of dyslipidemia was 30.3% in 2007 and 35.4% in 2008^36^,^37^. In our study, the prevalence of dyslipidemia also showed an increasing trend. However, data based on a Lithuanian middle-aged population showed a declining prevalence of dyslipidemia from 1985–2013^38^. Obesity is an independent risk factor of dyslipidemia, and the prevalence of dyslipidemia increases with BMI^39^,^40^. In our study, the rising trend in dyslipidemia was mainly attributable to the increased prevalence of high TG and low HDL-C levels, but not to high LDL-C. The prevalence of high LDL-C in this study decreased significantly in both men and women, and a similar result was also reported in a previous study, which showed a significant 5.7% decrease in LDL-C levels in adults aged 35–65 years from 1996 to 2007^41^. The variation in LDL-C levels may be explained by subjects’ use of lipid-lowering drugs^41^.

In this study, we observed a significant increase in the prevalence of NAFLD in both genders. The global prevalence of NAFLD is reported to be 25.24%, with the highest prevalence occurring in the Middle East and South America and the lowest in Africa^42^. NAFLD is strongly associated with obesity, in particular with visceral fat and insulin resistance, and with the increasing prevalence of obesity^43^. In addition, regarding clinical characteristics, NAFLD patients tend to be obese and to have insulin resistance and/or type 2 diabetes, hypertriglyceridemia, and hypertension^44^,^45^,^46^. NAFLD is increasingly recognized as a hepatic component of metabolic syndrome^47^. As the global epidemic of obesity fuels the metabolic conditions necessary for NAFLD, the clinical and economic burden of NAFLD are expected to become substantial. Our results are thus consistent with the increasing trends observed elsewhere.

In our study, the age-standardized prevalence of hypertension remained relatively stable from 2007 to 2013 in both genders (males: 38.10% vs. 38.63%; females: 33.04% vs. 33.01%, respectively), and this stability was consistent with a study in Turkey and another study in Italy^48^,^49^. The prevalence of hypertension did not show increasing trends as observed in all other diseases. The reason for this finding may be associated with a decrease in salt consumption. The Nutrition and Chronic Diseases in Chinese Residents study (2015) showed that the average daily salt consumption was 10.5 grams in 2012, indicating a decrease of 1.5 grams from the consumption in 2002^6^. However, some previous Chinese studies showed increasing trends of hypertension prevalence^13^,^50^. Data from the 24 geographically defined populations in the WHO Multinational MONItoring of trends and determinants in CArdiovascular disease (MONICA) Project showed that the age-adjusted prevalence of hypertension decreased in most and increased in only a few populations^51^.

The prevalence of hypertension increased with age in males and females in the two surveys. As age increases, a range of physiological changes occur, such as increased arterial stiffness, decreased renal salt excretion, declined renal function, and changes in the automatic nervous response to pharmacotherapy, which is used to manage hypertension^52^. However, the prevalence of the other diseases did not show the expected increasing trends with age.

Our results showed that men tended to have more obesity and obesity-related diseases than women in 2007 and 2013. The gender differences in these prevalences could be partially explained by men’s increased exposure to drinking and smoking.

This study is the first to assess the trends in the prevalence of obesity and obesity-related diseases using two cross-sectional health surveys in northeastern China with adjustments to minimize the differences in sample selection, measurements and case definitions.

It is important to acknowledge several limitations of our results. First, because of the nature of cross-sectional studies, causal relations could not be directly established. Second, we did not collect detailed information on diet, and we were thus unable to detect relationships between diet and the prevalence of the related diseases. Third, we measured FBG and blood pressure at a single visit, which might have led to an over- or underestimation of the true conditions. Finally, other confounders, such as different nationalities, socioeconomic status, residential density, noise pollution, drug use, diet, and physical activity, may also have influenced the results.

In conclusion, the results of these two cross-sectional surveys demonstrate a high prevalence of obesity and obesity-related diseases in the general population in northeastern China. Moreover, the prevalence of obesity, overweight, diabetes, pre-diabetes, dyslipidemia and NAFLD increased significantly from 2007 to 2013 in both men and women, whereas the prevalence of hypertension remained stable. Obesity and obesity-related diseases are the main risk factors of cardiometabolic diseases and pose a serious threat to the health of the general population^1^,^32^,^46^,^53^. More than 80% of the global diabetes and cardiovascular disease burden is expected to occur in low- and middle-income countries such as China and India by 2025^54^. Thus, urgent action, optimal treatment approaches and appropriate public health strategies are needed to prevent and manage these diseases, with the ultimate goal of lowering the incidence of cardiometabolic disease.

## Materials and Methods

### Study population

Two independent population-based cross-sectional studies of obesity and obesity-related diseases were conducted in Dehui City in Jilin, China, one in 2007 and one in 2013. Dehui is located 81 km from Changchun, which is the largest city in the area. Most inhabitants in Dehui earn in the average income range. The fifth National Population Census of China (2000) showed that the population composition, namely the sex and age distribution, of Dehui inhabitants was similar to those of Jilin in general; furthermore, the Dehui City Comprehensive Development Index (which considers regional, economic, cultural and other factors) represents the average of Jilin Province. Therefore, Dehui City was selected to represent other areas in the province. The designs of both cross-sectional studies were similar. Survey participants were selected using a stratified, multistage cluster probability sampling method. The questionnaire-based study was supervised and assisted by the National Bureau of Statistics of China. The study was approved by the Ethics Committee of the First Hospital of Jilin University, and all subjects participating in the study provided their written informed consent. All methods were carried out in accordance with the approved guidelines. A total of 3,636 and 1359 subjects aged 20–75 years were enrolled in the 2007 and 2013 cross-sectional studies, respectively.

### Data collection

All selected participants completed a standardized medical history and lifestyle questionnaire and underwent a comprehensive health examination according to routine procedures. A trained interviewer conducted face-to-face interviews with all participants and provided assistance to participants who had difficulty completing the questionnaire.

The participants fasted overnight (between 12 and 14 h) prior to receiving a comprehensive medical examination that included waist circumference (WC), standing height, weight and blood pressure (BP) measurements; a liver ultrasound; fasting blood samples to assess biochemical variables including liver enzymes, lipids, glucose and other routine blood measurements; and hepatitis B surface antigen (HBsAg) and anti-hepatitis C virus (anti-HCV) tests. Body weight and height were measured with the participants barefoot and in light clothing. Body mass index (BMI) was calculated as body weight divided by standing height squared. WC was measured on the horizontal plane between the inferior costal margin and the iliac crest on the mid-axillary line. Resting blood pressure was measured twice at 2-min intervals using a standard mercury sphygmomanometer after a 10-minute rest. Systolic blood pressure (SBP) and diastolic blood pressure (DBP) were defined as the average of the two readings. If the two measurements differed by over 10 mmHg, blood pressure was measured a third time, and the average of the three measurements was used as the final measurement. Abdominal ultrasonography (US) was performed by trained experienced radiologists to detect the presence of fatty infiltration in the liver, and the same equipment was used across studies (180 ultrasound machine with a 3.5 MHZ probe (GE Health care, Wilmington, MA, USA)) to minimize procedure-related variability^55^. Blood samples were centrifuged at the examination location, and the sera were stored at −20 °C until being tested at the First Hospital of Jilin University.

### Disease definition

In this study, diabetes was defined as a fasting plasma glucose (FPG) level ≥ 7.0 mmol/L, a previous diagnosis by a physician, or the use of insulin or oral hypoglycemic agents. Pre-diabetes was defined as 5.6 mmol/L ≤FPG < 7.0 mmol/L^56^.

Hypertension was defined as an average SBP ≥ 140 mmHg, an average DBP ≥ 90 mmHg, previously diagnosed disease, and/or the use of antihypertensive medication, regardless of BP readings^57^.

According to the Chinese Working Group on obesity, BMI < 18.5 kg/m^2^ was considered underweight, 18.5 kg/m^2^ ≤ BMI < 24 kg/m^2^ was considered normal, 24 kg/m^2^ ≤ BMI < 28 kg/m^2^ was defined as overweight, and BMI ≥ 28 kg/m^2^ was considered obese^58^.

According to the criteria of the “Chinese Guidelines on the Prevention and Treatment of Dyslipidemia in Adults”, hyperlipidemia was defined by a physician’s diagnosis and/or abnormal blood lipids (total cholesterol (TC) ≥ 5.18 mmol/L, triglycerides (TG) ≥ 1.7 mmol/L, high-density lipoprotein cholesterol (HDL-C) < 1.04 mmol/L or low-density lipoprotein cholesterol (LDL-C) ≥ 3.37 mmol/L)^59^.

Individuals with the following criteria were defined as having NAFLD: 1) a mean ethanol intake <140 g/week for men and <70 g/week for women in the past month, 2) a negative HBsAg and anti-HCV result, 3) fatty liver based on US, and 4) no other liver disease^60^.

### Statistical analysis

All analyses were stratified by sex. The Kolmogorov–Smirnov test was applied to continuous variables to test for normality. Non-normal data were presented as the median and quartiles, and Mann-Whitney U test was used to assess the differences between the two groups. Categorical variables were expressed as frequencies (percentage) calculated with Pearson’s Chi-square test. The prevalence of obesity and obesity-related diseases was standardized by age (ASR) using direct standardization based on the population composition of the Sixth National Population Census of China (2010). For standardization, we divided the participants into three age ranges (20–44 years; 45–59 years; and 60–75 years); these age groups were in accordance with the criteria of the WHO in 2012, except for a minor change, in which subjects 75 years old were included in the third range^21^. We compared the ASRs between 2007 and 2013 with the following formula: 
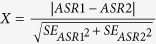
 (SE_ASR,_ standardization standard error). Data were analyzed using SPSS software version 20.0 (SPSS Inc., Chicago, IL, USA) with a significance level of P < 0.05 for all analyses.

## Additional Information

**How to cite this article**: Wu, J. *et al*. Six-year changes in the prevalence of obesity and obesity-related diseases in Northeastern China from 2007 to 2013. *Sci. Rep.* **7**, 41518; doi: 10.1038/srep41518 (2017).

**Publisher's note:** Springer Nature remains neutral with regard to jurisdictional claims in published maps and institutional affiliations.

## Supplementary Material

Supplementary Dataset 1

## Figures and Tables

**Table 1:** General characteristics of the study population in 2007 and 2013.

	2007(n = 3636)	2013(n = 1359)	P value
Sex (male)	1719(47.3%)	602(44.3%)	0.064
Age	45(36,55)	52(44,60)	<0.01*
BMI	23.89(21.48,26.45)	24.46(22.05,27.07)	<0.01*
WC	81(74,89)	83.5(77,91)	<0.01*
SBP	125(115,140)	130(118,146)	<0.01*
DBP	80(75,90)	82(78,90)	0.037*
TC	4.28(3.71,4.92)	5.00(4.00,5.00)	<0.01*
LDL-C	3.00(2.60,3.40)	2.65(2.12,3.18)	<0.01*
TG	1.22(0.83,1.90)	1.60(1.12,2.32)	<0.01*
HDL-C	1.3(1.2,1.6)	1.2(1.0,1.5)	<0.01*
FBG	4.86(4.48,5.33)	5.20(4.80,5.70)	<0.01*
ALT	18.0(13.5,26.9)	20.0(14.0,27.0)	0.022*
AST	21.3(17.6,26.0)	24.0(21.0,29.0)	<0.01*
Occupation (farmer)	1785(49.1%)	605(44.5%)	<0.01*

Result are expressed as the median (25^th^ quartile, 75^th^ quartile) or frequency (percentage).

BMI, body mass index; WC, waist circumference; SBP, systolic blood pressure; DBP, diastolic blood pressure; TC, total cholesterol; LDL-C, low-density lipoprotein cholesterol; TG, triglycerides; HDL-C, high-density lipoprotein cholesterol; FBG, fasting blood glucose; ALT, alanine aminotransferase; AST, aspartate aminotransferase.

^*^Indicates statistical significance.

**Table 2:** General characteristics of the study population by gender in 2007 and 2013.

	Male			Female		
	2007(n = 1719)	2013(n = 602)	P value	2007(n = 1917)	2013(n = 757)	P value
Age	45(36,55)	53(45,61)	<0.01*	46(37,55)	51(44,60)	<0.01*
BMI	24.17(21.73,26.71)	24.95(22.53,27.32)	<0.01*	23.59(21.53,26.22)	24.18(21.85,26.83)	<0.01*
WC	84(77,92)	85(78.4.92.2)	0.055	79(73,87)	82(76.5,89)	<0.01*
SBP	130(120,140)	130(120,144)	<0.01*	125(110,140)	130(116,148)	<0.01*
DBP	85(80,90)	85(80,90)	0.091	80(70,90)	80(74,90)	0.105
TC	4.36(3.78,4.97)	5.00(4.00,5.00)	<0.01*	4.22(3.64,4.86)	5.00(4.00,5.00)	<0.01*
LDL-C	3.00(2.60,3.14)	2.60(2.12,3.16)	<0.01*	2.97(2.50,3.40)	2.70(2.13,3.21)	<0.01*
HDL-C	1.3(1.1,1.5)	1.2(1.0,1.5)	<0.01*	1.4(1.2,1.6)	1.3(1.1,1.5)	<0.01*
TG	1.29(0.85,2.05)	1.57(1.06,2.39)	<0.01*	1.19(0.81,1.74)	1.63(1.16,2.25)	<0.01*
FBG	5.01(4.57,5.55)	5.30(4.90,5.80)	<0.01*	4.74(4.39,5.15)	5.10(4.80,5.60)	<0.01*
ALT	22.5(16.0,32.9)	22.0(17.0,30,0)	0.439	16.0(11.5,22.5)	18.0(13.0,24.0)	<0.01*
AST	23.2(18.8,28.2)	26.0(22.0,32.0)	<0.01*	20.1(16.9,23.8)	23.0(20.0,27.0)	<0.01*

BMI, body mass index; WC, waist circumference; SBP, systolic blood pressure; DBP, diastolic blood pressure; TC, total cholesterol; LDL-C, low-density lipoprotein cholesterol; TG, triglycerides; HDL-C, high-density lipoprotein cholesterol; FBG, fasting blood glucose; ALT, alanine aminotransferase; AST, aspartate aminotransferase.

^*^Indicates statistical significance.

**Table 3:** Comparisons of the prevalence of obesity, overweight, and obesity-related diseases between 2007 and 2013.

	Male				P value	Female				P value	P^+^ value	P^++^ value
	2007(n = 1719)		2013(n = 602)			2007(n = 1917)		2013(n = 757)				
	Crude	ASR	Crude	ASR		Crude	ASR	Crude	ASR			
Obesity	15.60%	15.82%	17.77%	19.41%	<0.01*	13.46%	13.18%	16.25%	18.77%	<0.01*	<0.01*	<0.05*
Overweight	36.60%	35.85%	41.69%	41.80%	<0.01*	32.39%	31.11%	36.20%	37.54%	<0.01*	<0.01*	<0.01*
Diabetes	6.98%	6.37%	11.79%	9.23%	<0.01*	5.16%	4.41%	8.59%	8.48%	<0.01*	<0.01*	<0.01*
Pre-diabetes	16.46%	16.77%	24.58%	23.49%	<0.01*	8.29%	8.10%	17.31%	16.49%	<0.01*	<0.01*	<0.01*
Hypertension	40.49%	38.10%	45.02%	38.63%	>0.05	35.89%	33.04%	41.22%	33.01%	>0.05	<0.01*	<0.01*
Dyslipidemia	54.40%	53.46%	64.80%	65.50%	<0.01*	45.30%	41.96%	60.60%	54.70%	<0.01*	<0.01*	<0.01*
NAFLD	23.79%	23.48%	40.53%	44.31%	<0.01*	18.83%	17.56%	47.42%	43.06%	<0.01*	<0.01*	<0.01*

ASR, age-standardized incidence rate (using the standard Chinese population in 2010); NAFLD, non-alcoholic fatty liver diseases.

P value, 2007 vs. 2013; p^ + ^value, males vs. females in 2007; p^ +  + ^value, males vs. females in 2013.

^*^Indicates statistical significance.

**Table 4:** The frequency of abnormal components in subjects with dyslipidemia between 2007 and 2013.

	Male					Female				
	2007		2013			2007		2013		
	Crude	ASR	Crude	ASR	P value	Crude	ASR	Crude	ASR	P value
TC	17.80%	17.12%	18.94%	15.98%	<0.01*	16.74%	14.84%	23.38%	18.91%	<0.01*
TG	33.86%	33.49%	44.35%	43.49%	<0.01*	26.66%	24.73%	47.29%	41.77%	<0.01*
LDL-C	28.50%	26.76%	18.11%	15.47%	<0.01*	27.49%	24.57%	20.21%	16.48%	<0.01*
HDL-C	15.30%	15.58%	32.72%	34.96%	<0.01*	7.62%	7.71%	22.32%	22.96%	<0.01*

TC, total cholesterol; LDL-C, low-density lipoprotein cholesterol; TG, triglycerides; HDL-C, high-density lipoprotein cholesterol; ASR, age-standardized incidence rate (using the standard Chinese population in 2010).

^*^Indicates statistical significance.
